# A study on the “community-hospital-community” model of community nursing practice teaching for undergraduate nursing students

**DOI:** 10.1186/s12912-023-01550-z

**Published:** 2023-10-17

**Authors:** Yuqing Li, Jiaofeng Gui, Ying Wang, Xiaoyun Zhang, Haiyang Liu, Lei-lei Guo, Jinlong Li, Yunxiao Lei, Xiaoping Li, Lu Sun, Liu Yang, Ting Yuan, Congzhi Wang, Dongmei Zhang, Huanhuan Wei, Jing Li, Mingming Liu, Ying Hua, Lin Zhang

**Affiliations:** 1 Department of Graduate School, Wannan Medical College, 22 Wenchang West Road, Higher Education Park, Wuhu City, An Hui Province P.R. China; 2 Student health center, Wannan Medical College, 22 Wenchang West Road, Higher Education Park, Wuhu City, An Hui Province P.R. China; 3 Department of Surgical Nursing, School of Nursing, Jinzhou Medical University, No.40, Section 3, Songpo Road, Linghe District, Jinzhou City, Liaoning Province P.R. China; 4 Department of Occupational and Environmental Health, Key Laboratory of Occupational Health and Safety for Coal Industry in Hebei Province, School of Public Health, North China University of Science and Technology, Tangshan, Hebei Province P.R. China; 5 Obstetrics and Gynecology Nursing, School of Nursing, Wannan Medical College, 22 Wenchang West Road, Higher Education Park, Wuhu City, An Hui Province P.R. China; 6 Department of Emergency and Critical Care Nursing, School of Nursing, Wannan Medical College, 22 Wenchang West Road, Higher Education Park, Wuhu City, An Hui Province P.R. China; 7 Department of Internal Medicine Nursing, School of Nursing, Wannan Medical College, 22 Wenchang West Road, Higher Education Park, Wuhu City, An Hui Province P.R. China; 8 Department of Pediatric Nursing, School of Nursing, Wannan Medical College, 22 Wenchang West Road, Higher Education Park, Wuhu City, An Hui Province P.R. China; 9 Department of Surgical Nursing, School of Nursing, Wannan Medical College, 22 Wenchang West Road, Higher Education Park, Wuhu City, An Hui Province P.R. China; 10 Rehabilitation Nursing, School of Nursing, Wannan Medical College, 22 Wenchang West Road, Higher Education Park, Wuhu City, An Hui Province P.R. China

**Keywords:** Community nursing, Community, Practice teaching, Model, Nursing students

## Abstract

**Objective:**

To improve the quality of community nursing teaching practice and cultivate undergraduate nursing students who meet the quality accreditation standards of our nursing profession, and to explore the establishment of an undergraduate nurse practice model.

**Methods:**

Using the methods of literature review, survey, expert consultation, and discussion, we established the steps and contents of community practice teaching for undergraduate nursing students, and implemented them for the students of Grades 2014, 2015, and 2016, and evaluated the “community-hospital-community” practice model through various forms, such as student self-evaluation, faculty evaluation, exit examination, and evaluation by certified experts.

**Result:**

A three-stage community nursing practice model of “community-hospital-community” was established for undergraduate nursing students. After three stages of practice, nursing undergraduates successfully passed the practical assessments and achieved excellent grades in each stage that met the requirements of the training program. In the first stage (community probation), community probation emphasizes a fundamental understanding of the community, using free clinics, health education, and home visits as entry points to effectively cultivate students’ job competence and proficiency in nursing operations and nurse-patient communication skills. In the second stage (internship in the hospital), through nursing internships in various systems, students are trained to integrate theoretical knowledge with practical skills and consolidate their understanding of fundamental knowledge, theory, and techniques. They are capable of preventing, diagnosing, intervening, and providing health education for common, frequent, urgent and critical complications in various clinical systems. They can formulate nursing plans and implement whole-person care. In the third stage (returning to the community for internship), students can master basic skills such as nursing operations and patient communication skills, and then they can enter the community internship.

**Conclusion:**

The community nursing practice model of “community- hospital- community” for undergraduate nursing students can systematically train undergraduate nursing students’ ability to work in the community.

**Supplementary Information:**

The online version contains supplementary material available at 10.1186/s12912-023-01550-z.

## Introduction

Due to the increasing demand for services, aging population, prolonged life expectancy and global financial challenges, the provision of health care services is becoming more and more complicated. The current model relies heavily on secondary health care rather than hospitals, resulting in a lot of waiting time and obstacles to access to health services [1–3]. Therefore, community nursing plays an increasingly prominent role in medical and health services. Community nurses [4] refer to professional nursing technicians who are engaged in community nursing work in community health institutions and other relevant medical institutions. They are the main force in community health care, and training community nurses is one of the important ways to improve the community medical service team and meet the community medical needs [5]. In 2016, China’s National Health and Family Planning Commission issued the National Nursing Career Development Plan (2016–2020), which explicitly proposed to accelerate the development of community nursing [6, 7], community nurses play an important role in the health of the population, and they are an important force to improve the level of community nursing services, and to gradually establish a long-term care service system through the extension of community nursing services to home care services.

The registration system of community nursing in foreign countries started early and is relatively perfect. Most foreign community nurses have bachelor’s degree, master’s degree or above [8], and the cultivation of high-level community nursing talents is more professional [9–12]. According to foreign research reports, the education of nursing students abroad emphasizes the multi-level and multi-field of curriculum [13]; Pay attention to the novelty and practicality of teaching methods, emphasize group participation, academic-community partnership mode, mixed learning mode and “Internet Plus teaching” mode to promote community nursing teaching [14, 15], so as to cultivate nursing students’ ability to solve problems independently and promote critical reflection. In terms of community clinical experience training, the United States and other countries often adopt the mode of interspersed practice for the arrangement of practical courses, and theoretical courses and practical courses are held at the same time [16]. In terms of continuing education for community nurses, the United States requires community nurses to regularly participate in continuing education and training for community nurses, so as to improve their nursing knowledge and skills and strengthen their comprehensive ability of community health management [17].

In contrast, although the school education of community nursing in China is basically the same as that in foreign countries, community nursing education in China started late, mainly in the vocational education of junior college and secondary school. At present, most community nurses are transferred from clinical hospitals, and they only have several years of working experience in some clinical departments, but they do not have the ability of community general nursing, so it is difficult to meet the needs of community nursing jobs [18–20]. However, the nursing graduates trained by the school are more inclined to work in hospitals and less likely to engage in community nursing [21]. Although community nursing has become a compulsory course of undergraduate nursing education in China. However, due to the late start of teaching, it is still in the stage of exploration and reform of teaching methods. Students’ concern and interest in community nursing learning are low [22, 23], and they lack initiative, and systematic research on practical teaching, and the practical teaching link is even weaker, which should arouse the common concern of schools and community nursing teaching practice bases.

The Code of Practice for Undergraduate Nursing Programs states that the teaching objectives of Community Nursing are: to complete the theoretical study and practical teaching of this course under the teaching guidance of professional teachers, to solidify students’ theoretical knowledge, to develop students’ abilities in community assessment and family health assessment, to improve students’ comprehensive clinical abilities, and to enable students to carry out unique community nursing and community health education.

Undergraduate nursing students need to go through four years study. Internships between classes in the third grade are an important part of nursing clinical education and an important way for nursing undergraduates to acquire professional knowledge, skills, attitudes, and behaviors that professional nurses must possess. This study aims to improve the post competence of undergraduate nursing students and constructs a new teaching model of “community-hospital-community”. In the first stage, the students went to the community for three times in the second semester of their junior year, each time for four hours; in the second stage, they went to the hospital from the sixth semester to the seventh semester for a total of 40 weeks. In the third stage, they returned to the community hospital again at the end of the seventh semester. It is hoped that this model can improve the teaching of community nursing, improve the post competence of undergraduate nursing students, and cultivate professional talents for the development of community nursing in China.

## Materials and methods

### Participants of study

Undergraduate nursing students in 2014, a total of 117 students. We started this model for the first time in 2017 and collected the data of junior students in that year (students in Grade 2014), and continued to verify this practice model every year since then.

### Implementation time of model

In the first stage (community probation), students enter the community for three times in the next semester of junior year (the fifth semester), with four hours each time; In the second stage, the students enter the hospital internship from the sixth semester to the seventh semester, for a total of 40 weeks. In the third stage, the students returned to the community for internship at the end of the seventh semester. Students in Grade 2015 and Grade 2016 also implemented this model in the same semester.

### “Community-hospital-community” teaching mode location and personnel

#### The sites in the first and third stages–Longjiang Community Health Service Center

**Fig. 1:**
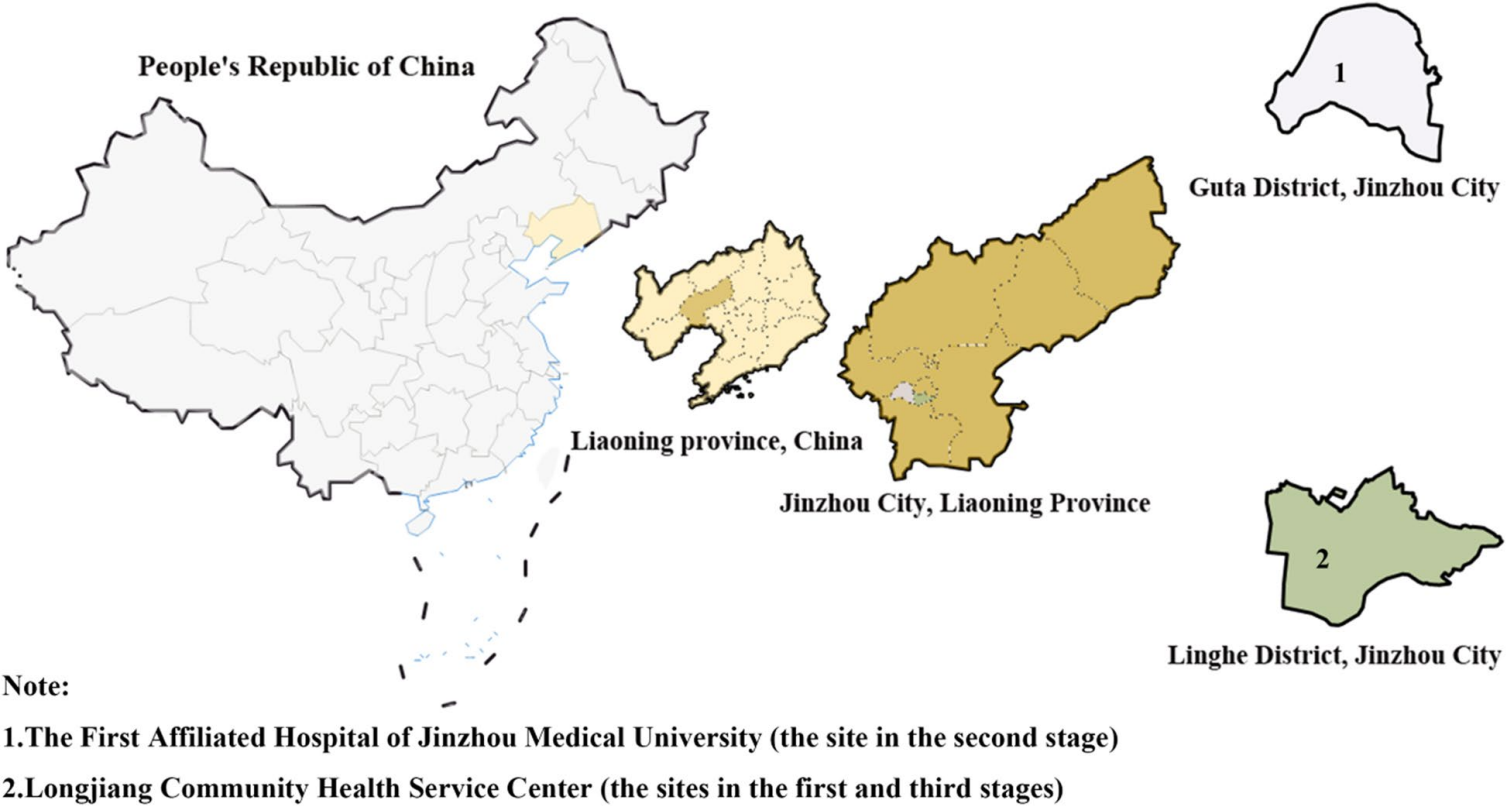
The location of practice

Longjiang Community Health Service Center, as a practical teaching center, is an excellent demonstration community located in Linghe District, Jinzhou City, Liaoning Province. The community covers an area of 1.36 square kilometers, with a population of 36,000. There are 7 communities in total, and the business area of the community center is more than 2,000 square meters. It has complete practical teaching facilities, including a general clinic, preventive health care department, planned immunization department (vaccination room), rehabilitation physiotherapy department, health education room, health information management room, and family planning department. The center adheres to the service concept of “based on the community, facing the society, giving priority to prevention and paying equal attention to the center”, strengthens the connotation construction, creates the characteristics of traditional Chinese medicine, and constantly improves and improves the service ability of “traditional Chinese medicine” in community health service centers. While fulfilling the “six-in-one” function of community health, it is guided by the needs of residents, strengthens the characteristics of traditional Chinese medicine, organically combines the development of traditional Chinese medicine with other work, and provides effective, positive, and integrated services for residents within its jurisdiction Figure 1 shows the location of the practice.

#### The sites in the second stage–the First Affiliated Hospital of Jinzhou Medical University

The First Affiliated Hospital of Jinzhou Medical University, founded in 1946, is a national large-scale comprehensive third-class first-class hospital and one of the three regional medical centers in Liaoning Province, which undertakes the tasks of medical treatment, teaching, scientific research, preventive health care, emergency and emergency treatment in Liaoning Province. As a teaching hospital, it undertakes the training of undergraduate college students and graduate students. The hospital regards the cultivation of medical humanities and professionalism as the basis of professional education; Clinical practice ability and clinical thinking training are the key points of professional training. Figure 1 shows the location of practice.

#### Syllabus revision experts

Practical teaching is led by the school’s Academic Affairs Office, which invites two experts from outside the school, four teaching managers, three full-time teachers from the teaching and research department, and four external teachers from the community to determine the content and steps of practical teaching through the Delphi back-to-back expert consultation method [24–26].

### Arrangement of community nursing teaching content and methods in the “community-hospital-community” teaching model

#### Theoretical study

The theoretical teaching of “Community Nursing” is arranged in the sixth semester of enrollment, and the third edition of Li Chunyu [27] is used as the theoretical teaching content, which is taught by the teachers of professional courses in the teaching and research department.

#### Practical teaching methods and steps

##### The first stage: community probation

The teaching content mainly includes 4 h of environment introduction, service content, service characteristics, service methods, health file establishment, etc., 4 h of health education, home visit, home care, etc. Community activities probation 3–4 times, using the community as the venue, during the school period to carry out 3–4 times of community volunteer medical and health education activities. The internship results will be evaluated by the internship report (Table 1).

**Table 1:** The first stage: community probation

Serial number	Items	Purpose and requirements	Practice content	Class hour
1	Overview of Community Health Service Stations and Community Nursing	1. Entering the community to understand the institutional setup, work nature, functions, and scope of community health service stations 2. Knowledge of the role function of community nurses, the main tasks and contents of nursing and health care services”	1. Organization setting, work nature, function, and scope of community health service station 2. Role function, nursing scope, and working methods of community nurses.	4
2	Community Population Health Assessment, Home Visiting, and Home Care	1. To be able to establish the general health care problems of the community and the health problems of special populations in the community, and to have the ability to collect and present relevant health information 2. To apply common community screening methods for health assessment and practice in establishing family health records 3. To be able to work effectively with community health service personnel and communicate effectively with community residents 4. To follow community nursing staff to conduct in-home newborn visits to understand the health status of newborns and families	1. Community health, community diet, physical exercise, and the correction of bad lifestyle 2. Maternal and child health care and child health care and planned immunization 3. Home care of common diseases in the community 4. Common health problems and nursing care of the elderly in the community 5. Community mental health and psychological counseling	4
3	Community Health Education	1. To be able to establish the topic and content of health education 2. To formulate the implementation plan of health education 3. To implement health education to guide community residents in personal and family health care. 4. To improve students’ language, communication, and health education skills through health education.	1. Health Education on common chronic diseases in the community 2.Health Education on Common Bad Habits of Community People 3. Knowledge of maternal and child health care	4

##### The second stage: internship in the hospital

Internship in a hospital is a mandatory requirement for nursing students in China to graduate. The school requires students to enter clinical practice for 40 weeks. It needs to be trained by departments of 13 major disease systems. The training scheme of each department is complete and feasible (Table 2).

**Table 2:** The second stage: internship in the hospital

Disease system	Practice department	Content	Internship duration(weeks)
**Respiratory system**	**Respiratory department**	**1. Theoretical knowledge** (1) Understand the pathogenesis and anatomical knowledge of common respiratory diseases. (2) Study the routine nursing care of respiratory diseases. (3) Master the nursing evaluation methods and evaluate the patients. (4) Master the indications and contraindications of arterial blood gas analysis, arterial blood collection, fiberoptic bronchoscopy, and thoracic puncture. (5) Familiar with the function, indications, indications, and contraindications of mechanical ventilation. **2. Practical objectives** Specialized operation: atomizing inhalation, arterial blood gas analysis, sputum aspiration. Basic nursing: oxygen inhalation, intravenous infusion, intramuscular injection, blood pressure monitoring, and ECG monitoring.	4
**Circulatory system**	**Department of Cardiovascular**	**1. Theoretical knowledge** (1) Understand the anatomy and physiology of the circulatory system. (2) Familiar with the common symptoms of circulatory diseases (3) Master the nursing evaluation, main nursing diagnosis/problems, and nursing measures of circulatory system diseases. **2. Practical objectives** Specialized operation: ECG monitoring, cardiopulmonary resuscitation, electric defibrillation. Basic operation: oxygen inhalation, intravenous infusion, intramuscular injection, and blood pressure monitoring.	2
**Blood system diseases or rheumatism immune system**	**Blood purification center or rheumatology immunology department**	**1. Theoretical knowledge** (1) Understand hematopoietic organs and hematopoiesis. (2) Understand the blood composition and physiological function of blood cells. (3) To master the definition and nursing diagnosis of anemia and be familiar with its clinical manifestations. (4) Understand the causes of common symptoms of rheumatic diseases. (5) Familiar with the characteristics of rheumatic diseases and nursing points. (6) To master the nursing evaluation, diagnosis, and measures of common symptoms of rheumatic diseases. **2. Practical objectives** Specialized operation: oral care, intravenous transfusion, postoperative nursing after bone marrow puncture. Basic operations: oxygen inhalation, ECG monitoring, intravenous infusion, intramuscular injection, blood pressure monitoring, and other operations.	2
**Digestive system**	**Digestive Department**	**1. Theoretical knowledge** (1) Understand the structure and function of the digestive system. (2) familiar with the pathogenesis of common diseases of digestive system. (3) Master the common symptoms of digestive system diseases and nursing measures. (4) Master the inspection methods and nursing skills of digestive system. **2. Practical objectives** Specialized operation: gastrointestinal decompression, nasal feeding, enema Basic operations: intravenous infusion, oral care, oxygen inhalation, intramuscular injection, ECG monitoring, etc.	4
**Urinary system**	**Urology urological department**	**1. Theoretical knowledge** (1) Understand the physiological function of urinary system and the main renal endocrine function. (2) Familiar with the relationship between the structure and function of urinary system and diseases (3) Master the principles of prevention and treatment of urinary system diseases and individualized nursing evaluation. **2. Practical objectives** Specialized operation: urethral catheterization, nursing with double J tubes, perineal nursing, and replacement of drainage tubes. Basic operations: oxygen inhalation, intravenous infusion, ECG monitoring, intramuscular injection, blood pressure monitoring, etc.	2
**Genital system**	**Gynaecology and obstetrics**	**1. Theoretical knowledge** (1) Understand the pathogenesis of reproductive system diseases. (2) Be familiar with the defense function of female reproductive organs. (3) To master the transmission route, secretion characteristics and treatment principles of vaginitis. **2. Practical objectives** Specialized operation: perineal scrubbing, catheterization Basic operation: intravenous infusion, ECG monitoring, intramuscular injection, and oxygen inhalation.	4
**Mental sickness**	**Psychiatry department**	**1. Theoretical knowledge** (1) Understand the nursing observation and daily life nursing of mental patients. (2) Familiar with the characteristics of mental patients’ contact and communication and the methods to promote effective communication. (3) Familiar with the common symptoms of mental patients and the prevention and nursing of crisis state. (4) Master the methods of basic nursing and organizational management of mental patients. **2. Practical goals (none)**	2
**Nervous system diseases**	**Neurology or neurosurgery**	**1. Theoretical knowledge** (1) Understand the classification, anatomical structure and function of peripheral nervous system and central nervous system, and the contents and steps of nursing evaluation for patients with nervous system diseases. (2) Familiar with the mechanism of action and pathophysiological changes of the nervous system, and the purpose and significance of common examinations of the nervous system. (3) Make a comprehensive evaluation of patients with nervous system diseases by using the knowledge learned. **2. Practical objectives** Specialized operation: evaluation of swallowing function and muscle strength. Basic operation: intravenous infusion, ECG monitoring, intramuscular injection, and oxygen inhalation.	4
**Motor system diseases**	**Neurology or geriatrics**	**1. Theoretical knowledge** (1) Understand the etiology and pathogenesis of Parkinson’s disease. (2) To master the clinical manifestations, treatment points and nursing care of Parkinson’s disease. **2. Practical objectives** Specialized operation: evaluation of muscle strength Basic operation: intravenous infusion, ECG monitoring, intramuscular injection, and oxygen inhalation.	2
**Metabolic endocrine system diseases**	**Endocrinology department**	**1. Theoretical knowledge** (1) Understand the relationship between the structure and function of endocrine system and diseases, (2) Familiar with the physiological functions of endocrine glands and the functions of main endocrine hormones. (3) Familiar with the principles of prevention and treatment of endocrine diseases and the nursing evaluation of specialized subjects. (4) Master the nursing routine of endocrine diseases. **2. Practical objectives** Specialized operation: insulin pen injection, installation and injection of insulin pump, blood sugar monitoring. Basic operation: intravenous infusion, ECG monitoring, subcutaneous injection, and oxygen inhalation.	2
**Operating room nursing**	**Operating room**	**1. Theoretical knowledge** (1) Understand the characteristics of the operating room building layout, the treatment methods of special infection surgery, the common surgical positions, and the management of clean operating room. (2) Familiar with the operating room rules and regulations, all kinds of fabrics, dressings, sets, cleaning, disinfection, sterilization, and maintenance methods of all kinds of items, and the clinical use of stitches and sutures. (3) To master the operating principle of aseptic technique, the operating room environment and the preparation of surgical materials, the names, functions, and usage of commonly used surgical instruments, the working procedures and responsibilities of hand-washing nurses and visiting nurses, the treatment methods of instruments, cloth, dressings and dirt after operation, and the precautions for correctly sending samples. **2. Practical objectives** Specialist operation: disinfection of surgical hands, putting on and taking off sterile surgical gown, sterile gloves, catheterization.	2
**Pediatric nursing**	**Pediatric/Neonatal Department**	**1. Theoretical knowledge** (1) Understand the anatomical and physiological characteristics of children’s test scores in various systems. (2) Familiar with the etiology, clinical manifestations, laboratory examination and treatment principles of common diseases of newborns and children. (3) To master the nursing evaluation, nursing diagnosis, nursing measures and health education of common diseases of newborns and children. **2. Practical objectives** Specialist operation: the use of incubator, phototherapy, bathing, changing diapers, milk preparation, feeding method, baby enema. Basic operations: intravenous infusion, femoral vein puncture, oral administration, nasal feeding, and oxygen inhalation.	4
**Emergency and critical nursing**	**Emergency department or ICU**	**1. Theoretical knowledge** (1) Understand the layout and equipment of the emergency room. (2) Familiar with the scope of emergency triage, the requirements of nurses’ responsibilities, emergency measures, first-aid instruments, and applications. (3) Familiar with the evaluation, clinical manifestations, pathogenesis, treatment principles, and rescue measures of major organ failure. (4) Master general emergency measures and understand the application principles of first-aid drugs. (5) To master the observation points of patients with craniocerebral injury. **2. Practical objectives** Specialized operations: cardiopulmonary resuscitation, electric defibrillation, application of gastric lavage machine, arterial puncture, central venous pressure measurement, nasal feeding, catheterization. Basic operation: intravenous infusion, ECG monitoring, subcutaneous injection, and oxygen inhalation.	4

##### The third stage: returning to the community for internship

After 40 weeks of clinical internship, students will have 2 weeks of community internship teaching for a total of 10 days. On the day of entering the department, they will have an induction education, which includes: an overview of the community, the purpose and significance of the internship, the requirements and methods of the internship, the management system of the internship, and the final assessment system. The students were arranged to enter the department by the academic affairs department and were taught one-on-one or one-on-two by external teachers (Table 3).

**Table 3:** The third stage: returning to the community for internship

Internship Department	Number of days of internship	Internship content	Appraisal requirements
**Community Resident Health Records Management Section**	**Two days**	1. Welcome each new student to the department, familiar with the department environment, clear department layout, facilities, work content, and workflow. 2. Guide the students to understand the establishment and management of health records, the management process of the elderly, hypertension, diabetes, coronary heart disease, stroke records, and some notes. 3. Establish the “people-oriented” service concept, learn to respect, and care for chronic patients in practice, and strengthen nurse-patient communication. Cultivate good psychological characteristics, optimism, cheerfulness, modesty, and caution, get along well with colleagues, abide by the ethical duties of nursing, and serve patients wholeheartedly. 4. Household follow-up visits for chronic diseases in community families	Participate in at least one community nursing assessment, one health education, and one chronic disease management
**Department of Chinese Medicine Physical Therapy and Rehabilitation**	**Two days**	1. Focus on teaching the clinical application of appropriate techniques of traditional Chinese medicine (TCM), such as acupuncture, cupping, moxibustion, and scraping, to enhance students’ knowledge and understanding of traditional Chinese medicine. 2. To lead students to know about TCM and understand the efficacy and usage of common TCM. 3. Introduce the advantages of pediatric massage in treating pediatric diseases to students, and briefly introduce the basic health care methods of pediatric massage. 4. To lead the students to visit the health education base of TCM, publicize the culture of TCM, popularize the knowledge of TCM, and show the long history, scientific theory, and unique methods of TCM, so that the students can personally feel the charm of TCM culture. 5. To popularize the concept of preventing diseases and health care methods of TCM to students, impart knowledge of TCM constitution identification, and let students know the nine constitutions and maintenance methods of TCM.	
**General Practice Clinic**	**Two days**	1. Familiar with the concepts of primary health care, community health service, and community nursing, make correct and reasonable community nursing diagnoses and community nursing assessments, and be familiar with and understand the epidemiological characteristics of chronic diseases. 2. Familiar with the contents of community health services, understand what is the six in one, understand the importance of “prevention” in community health work, and understand the combination of health education and clinical care. 3. Familiar with the common diseases and illnesses in the community, familiar with basic nursing operations, strict requirements to master aseptic operations, venipuncture, intramuscular injection, oxygenation, nebulized inhalation, and other operating procedures, precautions, familiar with the preparation of common skin test drugs, positive determination criteria, anaphylaxis rescue measures, the performance of common adverse reactions, rescue and treatment measures (participate in health education). 4. Understand the basic drug system, the basic drug catalog, and zero-price sales of drugs.	
**Maternal and child health care room**	**Two days**	1. Focused on knowledge of pregnancy care, time, and content of prenatal checkups. 2. Master the communication skills with pregnant women and mothers. 3. Understand the growth and development pattern of children from 0 to 36 months, make assessments and give guidance and suggestions on the results of children’s checkups. 4. Master the content of postnatal visits and take internship students to households for postnatal visits.	Participate in at least one family visit
**Vaccination Room**	**Two days**	1. Familiar with the work content, responsibilities, and various work processes of the department; master the contact and communication methods with families and children. 2. Master child immunization procedures, techniques, precautions, and vaccine management requirements, and be familiar with the types and classification of infectious diseases. 3. Understand community infectious disease reporting and management procedures, and participate in community infectious disease supervision and flow transfer. 4. Master the theoretical basic knowledge and basic skills of this department, establish good professional ethics, cultivate a high degree of responsibility, and improve the ability to analyze and solve problems alone.	Participate in at least one preventive vaccination

*TCM *Traditional Chinese medicine

##### Personnel and system in three stages

The requirements of teachers needed in the three stages are shown in Table 4.

**Table 4:** Personnel and system in three stages

	Lead teachers	Lead teacher training	Teaching management system
**The first stage: community probation**	The teaching staff consists of four full-time teachers and two part-time community teachers. Full-time teachers are required to have master’s degree or above. In addition, community teachers need to have intermediate or above titles and have more than ten years of work experience. They need to be loyal to the nursing cause, have a solid level of theory and skill operation, and have participated in general nursing job training, backbone training, and teacher training.	We set up a special section for teaching and learning, with the deputy director of the community service center as the head of the section. Introduced 3 undergraduates in clinical medicine and Chinese medicine; 5 people studied in provincial and municipal tertiary hospitals; participated in various academic activities and 116 provincial and urban training; 7 people participated in general practitioner transfer training; 4 people participated in provincial general practitioner backbone teacher training; participated in more than 10 times of teaching training organized by schools and nursing colleges; 5 times of outbound nursing teaching training.	Formulate teacher teaching system (clinical teaching system, teaching standard, lesson preparation standard), teacher management system (teacher selection and management, teaching director’s responsibility, teaching teacher’s responsibility, etc.), and nursing student management system (management system, leave and leave cancellation system, assessment methods, etc.).
**The second stage: an internship in the hospital**	Teachers are nurses in departments corresponding to each system. They need to have a college degree or above, have the title of the nurse in charge, and work in the hospital for more than 5 years; Or have a bachelor’s degree or above, the title of nurse practitioner, and have worked in clinic for more than 3 years.	The part-time teachers in the hospital are selected by the nursing department, and the training teachers need to have college education or above, the title of nurse in charge and more than 5 years of clinical work; Or have a bachelor’s degree or above, a nurse’s title, and clinical work for more than 3 years. The employment period is 2 years, and they need to participate in daily rounds, training, going out for further study, and other activities.	Formulate teacher teaching related systems (clinical nursing teaching system, clinical nursing practice teaching standard, clinical nursing practice lesson preparation standard), teacher management system, and nursing student management system (clinical nursing student management system, clinical nursing student leave application system, clinical nursing student assessment method, etc.).
**The third stage: returning to the community for internship.**	The team of lead teachers consisted of two full-time faculty members from the School of Nursing and seven external faculty members from the community. The full-time teachers of the nursing school are mainly responsible for theoretical teaching and some practical teaching, and the external teachers of the community are all professional and technical personnel who have been working for more than 10 years.	We set up a special section for teaching and learning, with the deputy director of the community service center as the head of the section. Introduced 3 undergraduates in clinical medicine and Chinese medicine; 5 people studied in provincial and municipal tertiary hospitals; participated in various academic activities and 116 provincial and urban training; 7 people participated in general practitioner transfer training; 4 people participated in provincial general practitioner backbone teacher training; participated in more than 10 times of teaching training organized by schools and nursing colleges; 5 times of outbound nursing teaching training.	Formulate teacher teaching system (clinical teaching system, teaching standard, lesson preparation standard), teacher management system (teacher selection and management, teaching director’s responsibility, teaching teacher’s responsibility, etc.), and nursing student management system (management system, leave and leave cancellation system, assessment methods, etc.).

“Full-time teachers” mainly attend classes in schools, and only go to hospitals or communities to attend classes when they receive teaching assignments; “Part-time teachers” are mainly engaged in clinical work in hospitals or communities, and also work in community or clinical teaching. “Professional and technical personnel” here refer to senior intellectuals who have certain knowledge and skills. In our manuscript, it represents medical personnel with more than ten years of medical experience. Due to the special characteristics of the grassroots community, one of the teaching teachers is a nursing major and six are clinicians, and all the general practitioners have been trained by the school system to lead the teaching and have been trained by general practitioners in Liaoning Province

#### Evaluation of teaching in three stages

##### The first stage: community probation

The probation teaching evaluation is in the form of probation report, and the score is given by the standardized requirements of our probation, with a full score of 100 points and a passing score of 60. See the Supplementary Material 1 for the scoring form (Supplementary Material 1: Probation report).

##### The second stage: internship in the hospital

All departments of the hospital make a final evaluation after the students’ internship, and assess them according to the learning content (Table 2), which is divided into qualified and unqualified.

##### The third stage: returning to the community for internship

Table 5 is the assessment content of community internship. The final written test score of community practice takes the average score of the test papers of five departments.The corresponding content is in Supplementary Material 2, 3, 4 and 5.(Supplementary Material 2: Daily work performance rating of students by supervising teachers; Supplementary Material 3: Home visits; Supplementary Material 4: Chronic disease community management program score; Supplementary Material 5: Examination questions for community internship).

**Table 5:** The assessment content of community internship

Serial number	Content	Percentage (%)
1	Attendance	10%
2	Daily work performance rating of students by supervising teachers	10%
3	Home visits	20%
4	Chronic disease community management program score	10%
5	Attendance examination	50%
Total score	100	

#### Graduation post-competency evaluation

Post competency [19, 28] refers to the abilities and qualities that a person needs to perform successfully in a particular position or occupation. It includes an individual’s knowledge, skills, experience, and abilities. Simply put, it is the qualification and ability to assume the post. China’s undergraduate nursing education focuses on the cultivation of professional talents, and job competence after entering the workplace is particularly important, not only to test the professional skills of nursing students but also to reflect whether a school’s training program is reasonable and feasible.

The unstructured interview method [29–31] and semi-open-ended questionnaire were used to interview and survey the study participants. The questionnaire mainly included 7 major topics such as community nursing knowledge and skills, observation and judgment, interpersonal communication and cooperation, empathy and responsibility, role awareness and transition, self-behavior and cognition, research, and innovation ability, etc., in which each topic contained 10 sub-questions. Each theme contains 10 sub-questions, using a 4-point scale of “strongly agree”, “agree”, “disagree” and “strongly disagree”, with scores of 4, 3, 2, and 1, and the total score was standardized by a percentage system to calculate the post competency [32, 33].

### Learning goals

From the perspective of KAP [34] (Knowledge, Attitude, and Practice), the learning goals of this study are as follows. Familiar with the workflow of community residents’ health file management department, traditional Chinese medicine physiotherapy rehabilitation department, general clinic, maternal and child health care department, and vaccination department, learn the establishment and management methods of health files, understand the efficacy and usage of common Chinese medicines, be familiar with common diseases and frequently-occurring diseases in the community, and master the health care knowledge of women and children and the theoretical knowledge of children’s immunization. Establish a “people-oriented” service concept and implement the belief in humanistic care. Master basic nursing operations (intramuscular injection, vaccination, oxygen inhalation, drug configuration for skin test, etc.), publicize the knowledge of traditional Chinese medicine and the importance of primary health care, participate in a family visit, participate in a community nursing evaluation, a health education, and a chronic disease management. Finally, to achieve the improvement of post competence and course performance. Finally, to improve the understanding of the community nursing course, and improve the post competence.

### Teaching quality control and evaluation

#### Personnel organization setting

**Fig. 2:**
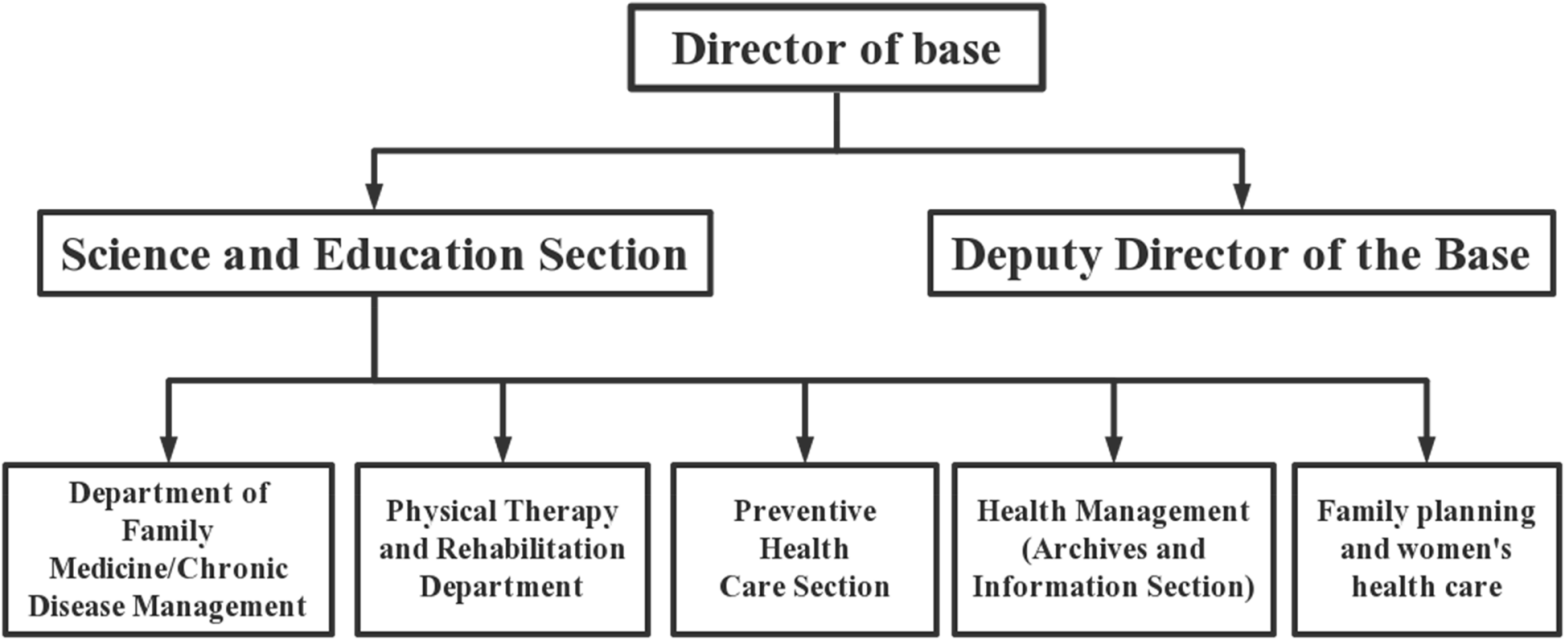
Organizational chart of teaching (Longjiang Community Health Service Center)

**Fig. 3:**
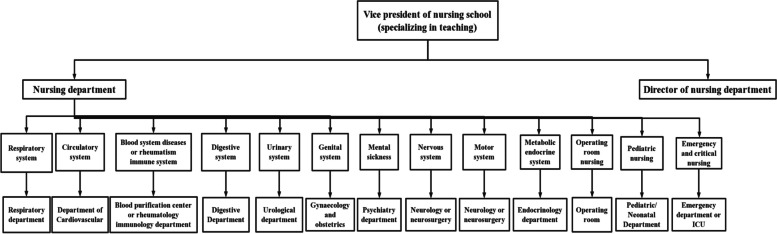
Organizational chart of teaching (The First Affiliated Hospital of Jinzhou Medical University)

Organizational chart of teaching as shown in Figs. 2 and 3. Figure 2 shows the teaching structure of Longjiang Community Health Service Center, in the first and third stages. Figure 3 shows the teaching structure of the first affiliated hospital of Jinzhou Medical University in the second stage of practice.

## Results

We invited Professor Zhao Yue from the School of Nursing of Tianjin Medical University and Professor Guo Guifang from the School of Nursing of Peking University as community nursing experts to visit and view the field. Both experts eventually responded favorably to this aspect of community practice teaching. In addition to the field investigation of several experts, we also have four evaluation indicators, namely, probation score, internship score, post competence, and degree satisfaction of internship (Table 6).

### Probation score

The probation scores of students in Grade 2014, Grade 2015 and Grade 2016 are 92.4±4.9, 93.6±5.1 and 94.6±3.6 respectively.

### Results of the third stage

All students passed the practical assessments in the third stage.

### Internship score

The internship scores of students in Grade 2014, Grade 2015 and Grade 2016 are 87.5 ± 6.8, 89.5 ± 7.4, and 88.3 ± 8.2, respectively. Compared with the scores of students in Grade 2013 who did not adopt this mode of internship (78.4 ± 5.7), they all improved.

### Post competence

After the practice, the scores of students’ post competence were all improved (87.6 ± 6.2 VS 91.2 ± 8.6/88.3 ± 5.7 VS 93.5 ± 7.9/86.2 ± 7.1 VS 94.4 ± 7.4), and the difference was statistically significant (*P* < 0.05).

### Degree Satisfaction of internship

After the internship, students and teachers rated their satisfaction with the learning process, and all evaluations were anonymous. Likert’s five-level scoring method was adopted, from very dissatisfied to very satisfied [35]. Satisfaction = (Satisfied + Very Satisfied)/Total *100%.

**Table 6:** Comparison of various indicators of probation and internship

Stage	Item		Grade		
			2014	2015	2016
The first stage	Probation score		92.4 ± 4.9	93.6 ± 5.1	94.6 ± 3.6
The second stage	qualified or unqualified		qualified	qualified	qualified
The third stage	Internship score		87.5 ± 6.8	89.5 ± 7.4	88.3 ± 8.2
	Post competence	Before internship	87.6 ± 6.2	88.3 ± 5.7	86.2 ± 7.1
		After internship	91.2 ± 8.6	93.5 ± 7.9	94.4 ± 7.4
		*P*	<0.05	<0.05	<0.05
	Degree Satisfaction of internship	Evaluation of students	113/115(98.26%)	124/127(97.64%)	119/121(98.35%)
		Evaluation of teachers	107/115(93.07%)	118/127(92.91%)	116/121(95.86%)

## Discussion

In our research, the results showed that nursing undergraduates have successfully passed the three stages of study and assessment, and the three-stage teaching practice mode of “community-hospital-community” has achieved good results. Students’ satisfaction degree is at a high level, and their academic performance has also improved. The evaluation results of post competence before and after practice showed that there was a significant difference in the total score of post competence before and after practice teaching (*P* < 0.05). In addition, we invited Professor Zhao Yue from the School of Nursing of Tianjin Medical University and Professor Guo Guifang from the School of Nursing of Peking University as community nursing experts to visit and view the field. Both experts eventually responded favorably to this aspect of community practice teaching.

The nursing discipline in developed countries started early, developed quickly, and relatively mature, with the trend of integration and comprehensiveness, and focusing on the combination of theory and practice which has formed a more mature clinical practice skills training model [36]. The biggest difference between developed countries and our country in the undergraduate nursing students’ practical skills training mode is the difference in internship. To improve the practical skills of students, some foreign institutions advocate early contact with the clinic, with more arrangements for laboratory courses, apprenticeships, and internship courses. The United States attaches particular importance to the practical skills training of nurses, such as the University of Pittsburgh, from the first year of enrollment. For example, the University of Pittsburgh from the first year of enrollment began to contact clinical practice, in addition to the theoretical and practical courses during the school year, but also according to the objectives of the curriculum to arrange clinical practice, from the shallow to the deep, and gradually increase the practical skills of students [37]; Northeastern University, Boston, in the community nursing program focuses on the cultivation of “multidisciplinary skills”, the proportion of community nursing practice also Northeastern University in Boston focuses on the development of “multidisciplinary skills” in the community nursing program, and the proportion of community nursing practice is up to 50%, which plays a key role in the development of community nurses’ practice skills [38].

In addition, nursing schools abroad provide opportunities for students to participate in community nursing practice under the auspices of some organizations or in collaboration with academic nursing societies, to consolidate theoretical knowledge and develop the competencies required for community nursing. For example, the University of Beirut has a 16-week, 9-hours-per-week community practice program. In this program, students are required to make home visits to designated families for 2 h per week. The University of Missouri Sinclair School of Nursing has offered a 2-week community-based nursing practice abroad program for selected undergraduate students since 2011, in which students enhance their adaptability, cultural awareness, and problem-solving skills.

At present, most of the training of nursing students in China still adopts the traditional “three-stage” course distribution model, that is, including the basic professional and public courses in the early stage, the middle stage of the comprehensive nursing courses, and the late stage of the clinical internship in the hospital [39]. Although there are occasional short-term practicums in community hospitals near the school, they are mostly bystander-oriented with few hands-on opportunities. Moreover, in the past 20 years, we have neglected the irreplaceable status work of community nursing as a community health service, the development of community nursing is relatively late, and there is no systematic and comprehensive training of community nurses in community nursing knowledge and practical skills [40]. If we copy the foreign model, it is not in line with our actual situation, so we should change the strategy from the source of talent training.

The three-stage community nursing practice model of “community-hospital-community” we have constructed for undergraduate nursing students, with classroom apprenticeship, community activities, and graduation internship as the main axes, fosters the students’ concepts of serving the community and grass-roots level and establishes the concepts of Chinese and Western medicine of prevention as the mainstay of prevention and the treatment of future illnesses to cultivate undergraduate nurses with the basic ability of engaging in community nursing. Talents. The internship and activities focus on the basic understanding of the community, with free clinics, health education, and family visit as the entry point, and effectively cultivate students’ job competence. At the end of the internship in the hospital, when the students master the basic skills of nursing operation, nurse-patient communication skills, etc., they will be allowed to enter into the community internship, and arrange the internship for 2 weeks with the community functional department as the basic unit, and set up the graduation internship mode with the community functional department as the unit, and cultivate basic community nurses according to the content and requirements of the internship. The content and requirements of the internship training of community nurses in the basic work capacity, the training process are mainly rules and regulations to regulate the apprenticeship internship students, through the subjective and objective assessment of students, assessment focus on the development of students’ job competence. In addition, nursing teachers can optimize the construction of nursing training mode through practice. Firstly, professional teachers can continuously improve their teaching and research ability by guiding students in community internship activities, participating in nursing service, education and research in community nursing service centers; carrying out teaching reform, formulating opinions on strengthening the implementation of teaching methods reform and put forward implementation plans for education and teaching research; thirdly, optimize the curriculum system according to the needs of community nursing training.

Based on the above findings, we put forward the following suggestions. First, improve relevant policies and systems to improve the level of community nursing education. Second, change the teaching concept of community nursing and innovate the training mode of community nursing talents by using various teaching methods. Thirdly, the content and standard of teaching evaluation should be formulated to improve the quality of nursing teaching. Fourth, strengthen the construction of a practice base and cultivate professional comprehensive ability.

## Limitations

First, in the process of practice, we also encountered some problems: the community activities organization link is larger, we usually select weeks off, and discuss with the student management community clinic, and family visits, due to more people, some students cannot be present; community practice base personnel quality varies, community nursing practice teaching staff nursing origin personnel less, some community clinicians still need to strengthen the teaching training. Second, the leave request and cancellation system of students sometimes cannot be complied with, and the home visit/health education objects cannot be executed as planned due to unexpected circumstances, which need to be further improved. Third, the participants in this study are junior students who entered the school in 2014. The first time to collect data was in 2017, which is slightly far from the current time. But every year after that, we continue to track the data of new junior students, hoping that this model can help improve our grades and understand the course.

## Conclusions

Through the practice mode of “community-hospital-community”, our result shows that the graduation performance has improved compared with that of undergraduate nursing students in Grade 2014, Grade 2015, and Grade 2016. And the total score of post competence has also improved before and after practical teaching. The community nursing practice model of “community- hospital- community” for undergraduate nursing students can systematically train undergraduate nursing students’ ability to work in the community.

### Supplementary Information

**Additional file 1:** The community nursing probation record.

**Additional file 2:** Daily work performance rating of students by supervising teachers.

**Additional file 3:** Family visit (stimulation) assessment criteria.

**Additional file 4:** Community management program rating table for common chronic diseases.

**Additional file 5:** Examination questions for community internship.

## Data Availability

The data presented in this study are available on request from the corresponding author. The data are not publicly available due to privacy reasons.

## References

[1] Burke S, Barry S, Siersbaek R, Johnston B, Ni Fhalluin M, Thomas S (2018). Slaintecare - A ten-year plan to achieve universal healthcare in Ireland. Health Policy.

[2] McDarby G, Smyth B (2019). Identifying priorities for primary care investment in Ireland through a population-based analysis of avoidable hospital admissions for ambulatory care sensitive conditions (ACSC). BMJ Open.

[3] (WHO) WHO. Technical series on safer primary care: transitions of care. Geneva; 2016.

[4] Yuping X, Yiping C, Qing W (2015). Discussion on the responsibilities of community nurses. Shanghai Med.

[5] Yanping Z, Jiajia X (2014). Study on the work function and training status of general nurses. Shanxi Med J.

[6] Commission TNHaFP (2017). National nursing development plan (2016–2020). Chin Nurs Manag.

[7] Jingyu D, Aihong W, Haiyan Y (2021). Application of a constructivism-based flipped classroom in teaching community nursing to undergraduate nursing students. Tianjin J Nurs.

[8] Meizhen L, Qiongying S, Meizhu P, Xiaohe J, Simei Z (2015). Problems and countermeasures of community nursing development under the family doctor service model. Hebei Med.

[9] Qi Z, Shanyu W (2018). Analysis of the current situation of community nursing personnel training at home and abroad. Chin Health Care Nutr.

[10] Xiaoxin L, Lezhi L (2016). Construction of competency index system of blood purification specialist nurses. Nurs Res.

[11] Arita K, Ryu H (2013). A comparison of trends in research into home care services in Japan and Korea. BMC Nurs.

[12] Chen CM (2013). Community health nursing: essential education elements. J Nurs.

[13] Alman BA, Ferguson P, Kraemer W, Nousiainen MT, Reznick RK (2013). Competency-based education: a new model for teaching orthopedics. Instr Course Lect.

[14] Joyce BL, Harmon MJ, Johnson RGH, Hicks V, Brown-Schott N, Pilling LB (2019). Using a quality improvement model to enhance community/public health nursing education. Public Health Nurs.

[15] Ezeonwu M, Berkowitz B, Vlasses FR (2014). Using an academic-community partnership model and blended learning to advance community health nursing pedagogy. Public Health Nurs..

[16] Hong W. Comparative study on the development of Chinese and foreign nursing higher education [D]. Chongqing Medical University; 2009.

[17] Hemingway A, Aarts C, Koskinen L, Campbell B, Chassé F (2013). A European union and Canadian review of public health nursing preparation and practice. Public Health Nurs.

[18] Qing L, Lingmin F, Leiyu F (2018). Construction of evaluation index system for competency of community nurses. Chin Nurs Res.

[19] Cuiling S, Lei S, Xinmei C, Xiuying X (2022). Evaluation of the effect of community nursing curriculum reform based on post competence. Nurs Pract Res.

[20] Xiaojian J, Guoping H (2010). Enlightenment of foreign community nursing system on the development of community nursing in China. Chin Gen Pract.

[21] Rui Z (2014). Thinking about the status quo and development of community nursing. Med Inform.

[22] Jianli B. Design and application research of Presentation-Assimilation-Discussion (PAD) class teaching model in community nursing teaching.[D] Shandong University of Traditional Chinese Medicine; 2018.

[23] Lin T, Suzhen L, Lan F (2020). Advances in research on practice teaching of general practice and community nursing at home and abroad. Chin J Social Med.

[24] Okoli C, Pawlowski SDJI (2004). Management: the Delphi method as a research tool: an example, design considerations and applications. ScienceDirect.

[25] Han L, Hao Y, Shujin Y (2012). Study on Delphi method for building communities practical teaching mode in nursing undergraduate in higher TCM colleges. Chin Nurs Res.

[26] Powell C (2003). The Delphi technique myths and realities. J Adv Nurs..

[27] Li C (2012). Community Health nursing.

[28] Siqing C, Liwen W, Yulian Z, Landi P (2009). A comparative study of community nursing practice models for nursing students in higher vocational colleges. Chin J Nurs Educ.

[29] Kase J, Doolittle B (2023). Job and life satisfaction among emergency physicians: a qualitative study. PLoS One.

[30] Kramer U (2014). Observer-rated coping associated with borderline personality disorder: an exploratory study. Clin Psychol Psychother.

[31] Mori F, Edquen SB, Espinoza ZEL, Salazar RS (2018). Competencies of the nurse in educational institutions: a look from educational managers. Rev Gaucha Enferm.

[32] Hou S, Sun J, Liu Y, Guifang G (2018). Application of community nursing course reform plan based on core of position competency. Chin Nurs Res.

[33] Xinqian Y (2014). Construction on competency evaluation Index System of TeachersIn Clinical practice of nursing undergraduates.

[34] XY D (2020). The application of the teaching model of knowledge, faith and action in the teaching of urological nursing students. China High Med Educ..

[35] Yunxia N, Suzhen L, Simin L (2014). Investgation of nursing students’ experiences and satisfaction in community practice. Chin J Pract Nurs.

[36] Lin C (2016). Bibliometric study on nursing mode at home and abroad.

[37] Li Y, Shufen Y, Shujie S, Xuemei Z, Ningning X (2014). Enlightenment of foreign nursing curriculum on higher nursing education in China. China High Med Educ..

[38] Nuo S, Ju N, Ruixing Z (2015). Comparison and practical exploration of community nursing education models at home and abroad. Henan Med Res.

[39] Xiaoxue W (2014). Study on teaching-training and course-setting ofInternational competent nursing undergraduates.

[40] Xuwen H, Yutao F, Guolian L, Meng N, Yunyun L, Lianhua Z, Yanmin Q (2019). Construction of Community Nurses’ practical skills Index System based on the Delphi method. Chin Nurs Res.

