# Supplementary material for: A study on the “community-hospital-community” model of community nursing practice teaching for undergraduate nursing students
Source: BMC Nurs. 2023 Oct 17;22:385. doi: 10.1186/s12912-023-01550-z (PMC10580528; doi:10.1186/s12912-023-01550-z)
Supplement: Supplementary file 1 — Additional file 1: The community nursing probation record. [file 12912_2023_1550_MOESM1_ESM.pdf]

Jinzhou Medical University
School of Nursing

The Community Nursing Probation Record Handbook

Name:

Student ID:

Grade/class:

Probation location:

Time:

Time：_____________location：_____________

teacher：______________professional ranks and titles：________________

Learning content:

study notes:

Recorder:

Teachers' overall evaluation of student performance:

Overall evaluation of students: excellent good qualified unqualified

Score (total score: 100 points):

Teacher signature：

Students' self-evaluation on the probation performance:

Overall evaluation of their own: excellent good qualified unqualified

Score (total score: 100 points):

Student signature：
