# Supplementary material for: A study on the “community-hospital-community” model of community nursing practice teaching for undergraduate nursing students
Source: BMC Nurs. 2023 Oct 17;22:385. doi: 10.1186/s12912-023-01550-z (PMC10580528; doi:10.1186/s12912-023-01550-z)
Supplement: Supplementary file 2 — Additional file 2: Daily work performance rating of students by supervising teachers. [file 12912_2023_1550_MOESM2_ESM.pdf]

Community Nursing Students in Longjiang Community Health Service Center

(Evaluation of Students)

**Name**		**Departme nt**		**Course title**		
**Teaching time**				**The teaching site**		
Teaching class						
**Item**	**Evaluating indicator**				**Total points**	**Score**
**Attitude to learning**	1. Students are full of spirit, active in the internship atmosphere, and can actively participate in the teaching activities organized by teachers				10	
	2. Students can respect teachers, listen carefully, think actively, speak enthusiastically, and take notes carefully				10	
	3. Students' attendance condition is good, and the class is orderly				10	
	4. Students are neatly dressed and meet the dress requirements of nurses				10	
**Internship behavior**	5. There is no slapping, looking at mobile phones, listening to music and other behaviors unrelated to study				10	
	6. Actively participate in the internship activities and follow the arrangement of teachers				10	
	7. Solid theoretical knowledge, able to apply the classroom theoretical knowledge to the internship practice				10	
	8. Be good at communicating with the trainees or staff members				10	
	9. Be good at finding the problems in the nursing internship and actively seeking answers				10	
	10. Be good at summarizing the gains and deficiencies in the internship				10	
**Total evaluation score**					**100**	
**Problems existing in teaching are:**			**Your advice:**			
