# Supplementary material for: A study on the “community-hospital-community” model of community nursing practice teaching for undergraduate nursing students
Source: BMC Nurs. 2023 Oct 17;22:385. doi: 10.1186/s12912-023-01550-z (PMC10580528; doi:10.1186/s12912-023-01550-z)
Supplement: Supplementary file 3 — Additional file 3: Family visit (stimulation) assessment criteria. [file 12912_2023_1550_MOESM3_ESM.pdf]

Jinzhou City, Linghe District Longjiang Street Community Health Service Center

Family visit (simulation) assessment criteria

Name：	Student ID：
Grade：	Class：
Major：	
Scenario title：	
**Items**	**Score**
1.Reflect the humanistic care for the interviewees and their families (20 points)；	
2.Actively talk with the interviewees and their families (20 points)；	
3.Inform the purpose and matters needing attention in detail before doing various inspections or operations (20 points)；	
4.The family visit begins to the end of all opportunities to educate the visitors (20 points)；	
5.Diet and medication notification and precautions tips (20 points).	
**Total points**	
