# Supplementary material for: A study on the “community-hospital-community” model of community nursing practice teaching for undergraduate nursing students
Source: BMC Nurs. 2023 Oct 17;22:385. doi: 10.1186/s12912-023-01550-z (PMC10580528; doi:10.1186/s12912-023-01550-z)
Supplement: Supplementary file 4 — Additional file 4: Community management program rating table for common chronic diseases. [file 12912_2023_1550_MOESM4_ESM.pdf]

Community management program rating table for common chronic diseases

Major: ______________________ Grade: ____________

Class: ______________________ Name: ____________

Location: ______________________ Time: ____________

Items	Total Score	Score
No leakage items	20	
Measure blood pressure 4 times or measure blood sugar 4 times	20	
To evaluate and formulate intervention programs and implementation measures	20	
Implementation summary	20	
Total score		
