# Supplementary material for: A study on the “community-hospital-community” model of community nursing practice teaching for undergraduate nursing students
Source: BMC Nurs. 2023 Oct 17;22:385. doi: 10.1186/s12912-023-01550-z (PMC10580528; doi:10.1186/s12912-023-01550-z)
Supplement: Supplementary file 5 — Additional file 5: Examination questions for community internship. [file 12912_2023_1550_MOESM5_ESM.pdf]

Linghe Community Health Service Center. Longjiang Street District

Family planning department test questions

Name: Score:

I. Multiple-choice questions (50 points in total, 10 points for each minor question)

1. At present, the most commonly used contraceptive method in China is ( ).

A. condom B. vaginal septum C. intrauterine device D. vaginal contraceptive E. oral contraceptives

2. The mistake about the follow-up time after IUD placement is ( ).

A. one month after placement B. three months after placement C. six months after placement D. nine months after placing E. twelve months after placing.

3. The best time for oral contraceptives is ( ).

A. before breakfast B. after breakfast C. at lunch D. before dinner E. after dinner.

4. Banned compound short-acting oral contraceptives disease is ( ).

A. breast cancer B. ovarian cancer C. endometrial cancer D. endometriosis e. trophoblastic tumor

5. Incomplete abortion after processing error is ( )

A. antibiotics B. oxytocin C. immediate uterine cleaning D. combined with infection should immediately curettage.

II. Medical record analysis questions (50 points in total, 12.5 points for each minor question)

<u>26 years old, female, 43 days, 3 days after induced abortion, sudden right lower abdominal pain:</u>

1. when collecting medical history should pay special attention to ask ( ).

A. vaginal bleeding B. the nature of lower abdominal pain C. whether there is syncope

D. whether there is villus E. in the curettage during operation. and the medication situation.

2. The key point of physical examination is ( ).

A. cervical lifting pain B. uterine mouth closure C. uterine size

D. posterior fornix tenderness E. whether there is a mass in the appendix.

3. The most valuable auxiliary examination for diagnosis is ( ).

A. pregnancy test B. B-ultrasound examination C. blood routine

D. urine routine E. uterine cavity examination

4. If the patient's urine HCG is more than 25U/L and blood WBC28*109/L, neutrophils are 85%, the possible diagnosis is ( ).

A. uterine foramen B. uterine aspiration insufficiency C. appendicitis D. pelvic inflammatory disease E. ectopic pregnancy

Linghe Community Health Service Center. Longjiang Street District

Family planning department test questions----Answer

I. Multiple-choice questions (50 points in total, 10 points for each minor question)

1、C　2、D　3、E　4、A　5、D

II. Medical record analysis questions (50 points in total, 12.5 points for each minor question)

1、D　2、A　3、B　4、D

Linghe Community Health Service Center, Longjiang Street District

Infusion center test questions

Name: Score:

I. Multiple-choice questions (20 points in total, 4 points for each question)

1. The drug of choice to rescue penicillin from anaphylactic shock is ()

A. promethazine hydrochloride B. oxyepinephrine C. epinephrine hydrochloride

D. Isoproterenol E. noradrenaline

2. When testing blood sugar, the most appropriate disinfectant for puncture site is ().

A. 75% alcohol B.0.5% chlorhexidine C. 2% chlorhexidine

D. 2% iodophor E. 0.5 iodine

3 The fastest route of administration is ().

A. oral B. external application C. inhalation D. subcutaneous injection

E. intravenous injection

4. The long tube in the closed thoracic drainage bottle is submerged under the liquid surface ()

A. 3-4cm B. 4-6cm C. 6-8 cm D. 8-10cm E. greater than 10cm

5 patients after brain surgery, no shock, coma, can take the lying position is ().

A. 10-20 degrees head height, foot slope position
B. 15-20 degrees head height, foot slope position
C. 15-20 degrees head low foot high slope position
D. 15-30 degrees head low foot high slope position
E. 15-30 degrees head height, foot slope position

II. Fill in the blanks (52 points in total, 4 points for each minor question)

1.The most common transfusion reaction (), the most serious transfusion reaction ( ).

2.The principle that nurses should follow when making the bed is: ( )( )( )( )

3.For infusion patients, the injection sequence is:( )( )( )( )

4.Fever reaction is a common reaction in the process of infusion, which is often caused by the input of ( ) substances.

5.The effective time of temporary doctor's advice is within ( )h, and it is generally executed ( ) times.

III. Short answer question(28 points in total)

1. Describe the principle, check the contents of seven pairs.(12 Points)

2. To describe the purpose of intravenous infusion.(16 Points)

Linghe Community Health Service Center, Longjiang Street District

Infusion center test questions----Answer

I. Multiple-choice questions

1-5: CAEAE

II. Fill in the blanks

1. Fever reaction, hemolytic reaction
2. First tidy up the head of the bed after the end of the bed, first tidy up the near side after the far side
3. First far after near, first small after big
4. Heat-causing
5. 24, 1

III. Short answer question

1. Describe the principle, check the contents of seven pairs

（1）Before, during the operation, and after the operation

（2）Bed number, name, drug name, concentration, dosage, time

2. To describe the purpose of intravenous infusion

(1) Correct electrolyte imbalance and maintain acid-base balance

(2) Supplement of energy and water content

(3) Input objects to treat the disease

(4) Increase in the blood volume to maintain the blood pressure

(5) detumescence of diuresis reduces intracranial pressure

Linghe Community Health Service Center, Longjiang Street District

Physical therapy rehabilitation department test questions

Name:　　　　　　　　　　　　　Score:

I. Fill in the blanks (20 points in total, 10 points for each minor question)

1. Moxibustion has curative effects on ( ), ( ), ( ), ( ), ( ), ( ), and all others.

2. Heat-sensitive moxibustion includes: ( ), ( ), ( ), ( ), ( ).

II. Multiple-choice questions (10 points in total, 5 points for each question)

1. Which of the following is not an index of sinus P wave ().

A. P wave time limit is 0.10 seconds.

B. P wave limb conduction voltage is 0.25 mv.

C. II, III and AVF leads are upright, and AVR is inverted.

D. The p-r interval is 0.12 seconds.

E. Lead II and III AVF are inverted and lead AVR is upright.

2. ECG can be seen that P wave widened, time > 0.11 seconds, and there is a notch to consider first ().

A. left atrial enlargement　B. left ventricular enlargement　C. right atrial enlargement

D. right ventricular enlargement　E. myocardial ischemia

III. Judgment questions (20 points in total, 5 points for each small question)

（　）1. Dazhui is located on the main vein, and its effect is to dredge meridians and activate collaterals.

（　）2. Sanyinjiao is the confluence of three Yang meridians.

（　）3. The effect of Zusanli is to treat all gynecological inflammation.

（　）4. Heat-sensitive moxibustion can't treat heat syndrome.

Ⅳ. Short answer question (50 points in total, 25 points for each small question)

1. What are the acupoints?

2. The effect of heat-sensitive moxibustion?

Linghe Community Health Service Center, Longjiang Street District

Physical therapy rehabilitation department test questions----Answer

I. Fill in the blanks (20 points in total, 10 points for each minor question)

1. heat disease, cold disease, real disease, deficiency disease, inside disease, surface disease

2. warm moxibustion, rotary moxibustion, meridian moxibustion, relay moxibustion, pecking moxibustion

II. Multiple-choice questions (10 points in total, 5 points for each question)

1.E 2. A

III. Judgment questions (20 points in total, 5 points for each small question)

1.× 2.× 3.× 4.×

Ⅳ. Short answer question(50 points in total, 25 points for each small question)

1. Acupoints are individualized, dynamic and sensitized disease body surface reaction parts, but also responsible for the regulation of human body function to achieve the purpose of disease prevention and treatment, needle and moxibustion stimulation part.

2. ① Activating blood circulation to remove blood stasis and expelling cold

② Prevention and health care to prolong life

③ Heat release, poison extraction, blood stasis and dispersing

Linghe Community Health Service Center, Longjiang Street District

Public health nursing exam questions

Name: **Score:**

I.Fill in the blanks (51 points, 3 points per space)

1. Community health services are centered on ( ), ( ) as the unit and ( ) as the scope.
2. The contents of personal health records include ( ), ( ), ( ) and other health service records.
3. The target of community health education includes not only the sick population, but also includes ( ), ( ), ( ) and ( ).
4. In the community health service work, the link to ensure the safety of medical care are ( ), ( ), ( ) and ( ).
5. Chronic disease refers to the general term of diseases with onset ( ), ( ) and ( ) with complex etiology and unclear pathogenesis, which need continuous treatment and care.

II. Judgment questions (25 points in total, 5 points for each small question)

( ) 1. The characteristics of the hypertensive population in China: low awareness rate, low treatment rate and high control rate.
( ) 2. The purpose of diabetes diet therapy is to limit carbohydrates.
( ) 3. Community nursing is an effective way to improve the treatment awareness and ability of the community population.
( ) 4. Health is not only no disease or no weakness, but the perfect state of physical and mental health and social adaptation.
( ) 5. Community nursing is a comprehensive discipline that combines nursing and public health theories to promote and maintain the health of the population.

III. Short-answer questions (24 points in total)

1. What are the roles of nurses in the establishment of residents' health records?(10 Points)

2. What are the public health services included?(14 Points)

Linghe Community Health Service Center, Longjiang Street District

Public health nursing exam questions---Answer

I.Fill in the blanks (51 points, 3 points per space)

1. Population health, family, and community

2. Basic personal information, health examination, and health management records of key groups

3. Healthy groups, high-risk groups, family members and caregivers of patients

4. Prevention, transformation, accident handling, and reflection

5. concealment, long course of disease, the disease is not cured

II. Judgment questions (25 points in total, 5 points for each small question)

1. × 2.× 3.× 4.√ 5.√

III. Short-answer questions (24 points in total)

1. ① Health Caregiver; ② Health planner; ③ Health Coordinator; ④ Health Educator; ⑤ Organization Administrator; ⑥ Nursing investigator; ⑦ Community Health spokesperson.

2. Public health services include:

(1) Management of health records for urban and rural residents.

(2) Health education.

(3) Vaccination.

(4) Health management of children aged from 0 to 6 years old.

(5) Maternal and maternal health management.

(6) Health management of the elderly.

(7) Health management of hypertensive patients.

(8) Health management of patients with type 2 diabetes mellitus.

(9) Health management of patients with severe mental illness.

(10) Reporting and handling of infectious diseases and public health emergencies.

(11) Health supervision and assistant management services.

Linghe Community Health Service Center, Longjiang Street District

Vaccination section test questions

Name: Score:

I. Multiple-choice questions (25 points in total, 5 points for each question)

1. Basal immunity is required to be completed within the ( ).

A. 7 years B. 1 year and a half C. 24 months D. 12 months

2. The interval between each dose of polio vaccine and DPT vaccine should be ( ) days

A.≥28 B.≤28 C.≥30 D.≤30

3. There are two doses of meningococcal group A vaccine, and the interval between the two doses should be

A. one month B. two months C. ≥ three months D. four months

4. The storage period of vaccination cards should be kept for not less than ( ) years after children reach the age of 7.

A. 3 B. 5 C. 7 D. 15

5. Which of the following statements is incorrect ()

A. the inoculation site should avoid scars, inflammation, induration, and skin diseases.

B. 20% tincture of iodine is prohibited for skin disinfection before inoculation, and 75% ethanol is used.

C. under the same inoculation route, the greater the dose, the better the immune effect.

D. disposable syringe after use shall not be back to the needle cap, should be destroyed after soaking disinfection, unified recycling, and destruction.

II. Fill in the blanks (50 points in total, 5 points for each minor question)

1. Inoculate local skin for disinfection. Use a sterile cotton swab dipped in 75% ethanol, and disinfect from ( ) to ( ), ( ) with a diameter of ≥( )cm. Inoculate immediately after drying.
2. The hepatitis B vaccination procedure is: children should be vaccinated at ( ), ( ) and ( ) respectively.
3. The object of family visit is ( ), and the first and second visit time is ( ) and ( ).

III. Short answer question (25 points in total)

1. What are the main contents of the report of abnormal reaction to vaccination? (15 Points)

2. What are the basic items included in the family visit package? (10 Points)

Linghe Community Health Service Center, Longjiang Street District

Vaccination section test questions---Answer

I. Multiple-choice questions

1.A 2.A 3.C 4.D 5.C

II. Fill in the blanks

1. Inside, outside, screw type, 5
2. Born 24 hours, One month, 6 months
3. Neonates and parturients at 7 days postpartum or three days after discharge, Seven days postpartum or three days after discharge, About a month after delivery

III. Short answer question

1. Name, sex, age, name, address of the child's guardian, name of vaccination, Time, time of inoculation, time and number of reactions, main clinical characteristics, Preliminary diagnosis and diagnosis unit, reporting unit, reporter, reporting time, etc.
2. Basic items include: stethoscope, blood pressure monitor, tape gauge, thermometer, baby weight Scales, flashlight, tongue pressing plate.
